# Pertussis: The Identify, Isolate, Inform Tool Applied to a Re-emerging Respiratory Illness

**DOI:** 10.5811/westjem.2018.11.40023

**Published:** 2018-12-05

**Authors:** Kristi L. Koenig, Jennifer Farah, Eric C. McDonald, Sayone Thihalolipavan, Michael J. Burns

**Affiliations:** * County of San Diego, Health & Human Services Agency, Emergency Medical Services, San Diego, California; † University of California Irvine, Department of Emergency Medicine, Orange, California; ‡ University of California, San Diego, Department of Emergency Medicine, La Jolla, California; § County of San Diego, Health & Human Services Agency, Public Health Services, San Diego, California; ¶ University of California Irvine, Department of Emergency Medicine and Division of Infectious Diseases, Orange, California

## Abstract

Pertussis, commonly referred to as “whooping cough,” is a highly contagious acute respiratory infection that has exhibited cyclical outbreaks throughout the last century. Although vaccines have provided some immunity, many populations, including infants and pregnant women, remain at risk for serious illness. Through the use of the novel “Identify, Isolate, Inform” (3I) tool, emergency department (ED) providers can readily recognize key symptoms of the disease and risk factors for exposure, thus curbing its transmission through early initiation of antimicrobial therapy and post-exposure prophylaxis. The three classic stages of pertussis include an initial catarrhal stage, characterized by nonspecific upper respiratory infection symptoms, which may advance to the paroxysmal stage, revealing the distinctive “whooping cough.” This cough can persist for weeks to months leading into the convalescent stage. Household contacts of patients with suspected pertussis or other asymptomatic, high-risk populations (infants, pregnant women in their third trimester, and childcare workers) may benefit from post-exposure prophylactic therapy. The Pertussis 3I tool can also alert healthcare professionals to the proper respiratory droplet precautions during contact with a symptomatic patient, as well as isolation practices until antimicrobial treatment is in progress. ED personnel should then inform local public health departments of any suspected cases. All of these actions will ultimately aid public health in controlling the incidence of pertussis cases, thus ensuring the protection of the general public from this re-emerging respiratory illness.

## INTRODUCTION

Pertussis, commonly referred to as “whooping cough,” is an acute respiratory illness that is highly contagious. *Bordetella pertussis*, a Gram-negative bacterium, travels via respiratory droplets infecting human hosts.^1^ Worldwide epidemics have occurred throughout history, prompting study and control measures, including the development of vaccines.^1^ However, even in vaccinated populations, pertussis demonstrates periodic outbreaks. For example, in 2010 California experienced a large outbreak that reached the highest incidence rates of the disease since 1947. This outbreak involved over 9,000 individuals and led to 10 infant deaths.^2^,^3^ In 2017 there were over 15,000 cases in the United States, with California reporting the highest number at 1,742 cases.^4^

Given this background, and following on previous work for Ebola virus disease, measles, Middle East Respiratory Syndrome, Zika, mumps, and hepatitis A, investigators developed a novel Pertussis Identify, Isolate, Inform (3I) tool for use by healthcare workers in the assessment and treatment of patients who may have pertussis (Figure).^5^–^10^ After an overview of the disease and critical information pertaining to transmission and treatment, we explain and present here the Pertussis 3I tool.

The presentation of pertussis varies widely, and can be affected by factors such as vaccination status and age. It is classically described as having three stages.^11^–^14^ After an initial incubation period of 7–10 days, the disease begins with the catarrhal stage, which has a duration of one to two weeks. This manifests as a mild cough with lacrimation and rhinorrhea. There may also be a low-grade fever. After the catarrhal stage, the patient may advance to the paroxysmal stage, which lasts two to four weeks. This is where the characteristic paroxysmal or “whooping cough” may occur, described as a grouping of multiple short coughs followed by a single, forceful inspiratory “whoop.” An audio example of this signature cough is available here: http://www.pkids.org/diseases/pertussis.html. This cough may be associated with emesis, cyanosis or even apnea.^15^ The third or convalescent stage is characterized by a persistent cough that can last from four weeks up to several months. This is why pertussis is known as the “100-day cough” in China.^16^

Older children, adolescents, and adults may report a nonproductive cough that is worse at night or feelings of a choking sensation. They likely will be asymptomatic between coughing episodes.^11^ Presenting symptoms may be nonspecific in both infants and older patients. Young infants may initially be afebrile with mild symptoms that rapidly progress to respiratory distress/apnea, hypoxia or seizures.^12^

### Risk Factors

Unvaccinated individuals, or those who have not yet completed the vaccine series, are the most at risk. This includes infants <six months of age, who are also at the highest risk for severe outcomes.^17^ However, even persons who have received the vaccine series lose their immune status within six to eight years of their last injection or 15 years after infection.^13^ Thus, remaining up to date with vaccination is imperative, especially when traveling abroad to areas with increased disease incidence.^18^ Additionally, household contacts and those considered high risk, who have known exposure, should receive treatment in the form of post-exposure prophylaxis (PEP) (see “Treatment” section).

### Diagnosis

Nasopharyngeal cultures, polymerase chain reaction (PCR) testing and serologic studies are available to confirm an infection with *Bordetella pertussis*, the causative organism.^11^ However, these tests offer varying levels of sensitivity and may not be obtainable in a timely fashion to confirm cases in the acute setting. Furthermore, other laboratory studies, such as a complete blood count (CBC), may be helpful in distinguishing causes for cough, but only in certain age groups (see “Differential Diagnosis” section). Imaging studies also provide limited information, as patients often do not demonstrate significant findings on chest radiograph. However, chest imaging may be helpful in assessing for superinfection.

**Population Health Research Capsule**

What do we already know about this issue?

*Pertussis, or whooping cough, a highly contagious respiratory illness, presents in cyclical outbreaks every few years*.

What was the research question?

*Investigators sought to modify the “Identify, Isolate, Inform” (3I) Tool for use in the identification and management of pertussis*.

What was the major finding of the study?

*A novel Pertussis 3I Tool is created for real-time application in managing patients presenting to the emergency department (ED)*.

How does this improve population health?

*The 3I Tool aids ED providers who play an essential role in identifying and treating this vaccine-preventable disease*.

### Complications and Special Populations

Severe and sometimes fatal pertussis-related complications can occur in certain groups. These include infants <12 months of age, particularly those <four months, as well as pregnant women who are also at risk of transmitting the disease to their newborn children.^17^,^19^ Often, patients become secondarily infected with another bacterial or viral infection. Neonates are especially at risk for apnea and hemodynamic instability (i.e., bradycardia, hypotension). Although rare, seizures and encephalopathy can also occur.^12^,^17^,^19^

### Transmission and Personal Protective Equipment

Pertussis has no known animal or environmental hosts.^1^ It travels from human to human via respiratory droplets from a cough or sneeze. Patients who have not yet started or completed the initial vaccine series are at greatest risk of becoming infected. If there is concern that a patient has pertussis, healthcare workers should place the patient in isolation and don personal protective gear for respiratory droplet precautions.^20^

### Prevention

In addition to protective measures to avert disease transmission, the most important preventative measure is vaccination. In 2018, the Centers for Disease Control (CDC) and Prevention’s Advisory Committee on Immunization Practices published the following vaccine recommendations:^21^

- Infants and young children: five-dose series of diphtheria and tetanus toxoids and acellular pertussis (DTaP) vaccines, with one adolescent booster dose of tetanus toxoid, reduced diphtheria toxoid, and acellular pertussis (Tdap) vaccine
- Adults: booster dose of Tdap (regardless of vaccine status)
- Pregnant Women: one-dose Tdap to be administered sometime during 27–36 weeks gestation (third trimester), regardless of previous receipt of Tdap
- Persons >11 years with close contacts of infants (e.g., parents, siblings, grandparents, child care providers, and healthcare providers): administer a single dose of Tdap if they have not previously received Tdap
- Healthcare personnel: administer a single dose of Tdap if they have not previously received Tdap

### Differential Diagnosis

Given the nonspecific nature of presenting symptoms, diagnosing pertussis can be challenging. In addition, the characteristic “whooping” cough is only appreciated in a minority of patients.^13^ Thus, other causes for similar complaints must also be considered, including upper respiratory infections or pneumonia. Clinicians should also contemplate asthma, bronchiolitis (respiratory syncytial virus) or adenovirus in the differential diagnosis for children.^13^ Of special note, in infants nearly all fatal cases of pertussis present with an extreme leukocytosis with lymphocytosis.^17^ Thus, obtaining a CBC may be helpful to distinguish between causes of cough in pediatric patients; however, its utility remains limited. Adults presenting with cough may have non-infectious causes for their symptoms such as chronic obstructive pulmonary disease, congestive heart failure, or gastroesophageal reflux disease.^22^ Foreign body aspiration is also possible in patients presenting with cough and is sometimes associated with cyanosis or apnea.

### Treatment

Suctioning and other airway management is a mainstay of management. As with other conditions, in the presence of hypoxia or respiratory distress, supplemental oxygen should be applied. Intravenous fluids may also be needed for treatment of dehydration.^19^,^23^ In addition to supportive care, antimicrobial treatment is recommended. Macrolides are the preferred treatment, which include azithromycin, clarithromycin or erythromycin. 19,23,24 For infants <one month of age, azithromycin is the preferred antibiotic.^14^,^19^ For patients who cannot tolerate these medications, and are >two months of age, trimethoprim/sulfamethoxazole is recommended.^19^,^23^,^24^

PEP is limited to certain groups (Table).^25^ These include household contacts of a pertussis case and high-risk populations. With regard to household exposures, even if these contacts are asymptomatic and/or current with immunizations, it is recommended they receive antimicrobial treatment within 21 days of cough onset in the index patient. High-risk groups include infants, women in their third trimester of pregnancy, caregivers or household contacts of infants, and anyone who works in or attends a childcare setting.^25^ Antibiotic selection and duration of treatment for either PEP or a confirmed case of pertussis are identical. Depending on the patient’s age and therapy of choice, treatment includes a 5–14 day course of a macrolide, with the treatment duration dependent on the macrolide chosen. In cases of PEP, treatment should be initiated within 21 days of exposure.^19^, ^23^

### Disposition

Although dependent on provider judgment, patients with mild to moderate disease can be safely discharged home to undergo antibiotic treatment, with careful attention noted to household contacts or other possibly exposed individuals. Hospital admission is recommended for neonates because they are at risk for apnea.^19^ Additionally, admission is recommended for patients <six months of age or who have a history of prematurity. Other symptoms to consider when determining need for hospitalization include inability to tolerate fluids or persistent dependence on supplemental oxygen.^13^,^19^,^23^ Admitted patients should be maintained in respiratory droplet isolation.^20^

## IDENTIFY, ISOLATE, INFORM

### Identify

Identification of two groups of patients is important: those who are symptomatic, and those who are asymptomatic but have been exposed. Both groups may benefit from treatment. Symptomatic individuals may present in any one of the three classic stages of pertussis, as discussed above. Some may be in the initial catarrhal phase, reporting mild upper respiratory symptoms, and others may have progressed into the paroxysmal phase, exhibiting the classic “whooping cough.” Other symptoms commonly reported include post-tussive emesis, cyanosis and apnea. Patients who have had a persistent cough for weeks, and perhaps months, may be in the convalescent phase. All of the aforementioned presentations may represent a patient with pertussis, making careful reviews of exposure history and immunization status essential. Importantly, those with previous vaccinations may present atypically and not exhibit classic features of pertussis.

Another important group to consider are those who deny symptoms, but report having been exposed to a person with confirmed pertussis. Pregnant women and infants are especially at risk; thus, review of this type of exposure is critical when deciding whether to initiate treatment. Patients are considered most contagious three weeks after the onset of the paroxysmal phase, where coughing spells are most prevalent. Thus, asking exposed patients when they were with the source patient could aid in assessing their individual risks.

### Isolate

If a symptomatic, and thus potentially contagious, patient has been identified, he or she should immediately be placed in droplet isolation.^20^ Healthcare personnel should also don personal protective gear for respiratory droplet precautions, irrespective of their own vaccine status. This includes donning a standard surgical mask. Patients are considered infectious from the beginning of the catarrhal stage until three weeks after the onset of the paroxysmal stage.^24^ Thus, isolation may be needed during this length of time. However, evidence suggests that those undergoing antibiotic treatment may no longer be contagious five days after initiating treatment.^24^,^26^

This timeline is even more important when one considers returning to school or work. With the former, the CDC and the American Academy of Pediatrics recommend children with pertussis refrain from attending school until they have completed five days of antibiotic treatment.^25^ However, since pertussis is often not definitively diagnosed, it’s unclear as to the true benefit of this exclusion, leading some public health authorities to adopt a more liberal policy of allowing children who have started, but perhaps have not yet completed, their antibiotic course to attend school. 25 Consultation with one’s local health department may assist with this decision. In summary, discretion must be taken when a patient is undergoing either inpatient or outpatient treatment, particularly when close contacts include infants or pregnant women.

Diagnosis should be confirmed with nasopharyngeal cultures, PCR testing and/or serologic studies. Test selection is based on the timeframe of symptoms.^27^ If the patient reports a cough of less than two weeks duration, both a culture and PCR should be performed. If the cough has been present for between two and four weeks, culture becomes less reliable, and thus PCR is recommended. Serology is the only reliable diagnostic tool after four weeks of symptoms. However, serology measures pertussis antibodies, and these levels may be affected by stage of the disease and vaccination status.^11^ Therefore, providers should consider these confounding factors when interpreting serologic studies.

Practitioners must pay close attention to use of the proper technique for obtaining a nasopharyngeal specimen, whether it is for culture or PCR testing. *B. pertussis* resides in the posterior nasopharynx. Therefore, the swab must be inserted past the anterior nare to ensure optimal collection.^11^ Cotton-tipped or rayon swabs should not be used, as they contain chemicals that can alter results; rather, a calcium alginate or polyester (e.g., dacron) swab affixed to a long metal shaft is indicated.^11^ A video depicting the proper technique for specimen collection is available on the CDC website at https://www.cdc.gov/pertussis/clinical/diagnostic-testing/specimen-collection.html.^28^

### Inform

If a pertussis case is suspected, healthcare workers should contact their local health department, as well as their hospital infection prevention department.^29^ Clinicians should also assess for household or other close contacts and provide them with appropriate education and follow up. Contacting public health agencies can occur through a number of channels, and may depend on local public health department policies. Providers should notify their local public health agency of any cases of suspected pertussis. This may include patients with paroxysms of cough, the classic inspiratory “whoop,” post-tussive emesis and/or apnea (for infants less than one year old). 29 Laboratories who identify confirmatory tests for pertussis should also report to local public health authorities. Local public health agencies can then forward their findings to state agencies, which will then share this information with the CDC through the National Notifiable Diseases Surveillance System (NNDSS).^30^ This chain of reported information allows for further investigation. Given the limitations of confirmatory testing, it becomes even more essential for healthcare workers to report clinically suspected cases of pertussis, so that health officials can conduct continued surveillance.

## CONCLUSION

CDC reports suggest the incidence of pertussis exhibits cyclical rises and falls.^31^ Therefore, there is an imminent need to routinely educate healthcare workers on its clinical features and epidemiologic properties so that they can promptly detect and appropriately manage pertussis cases. The novel Pertussis Identify, Isolate, Inform (3I) tool can aid emergency department staff in readily recognizing key symptoms of the disease and risk factors for exposure. The Pertussis 3I tool can also alert the healthcare workforce to the appropriate isolation protocols for use during contact with a symptomatic patient. With this added knowledge, healthcare workers can protect both themselves and others (especially infants and pregnant women) from contracting disease. Further, they can educate patients, in addition to exposed individuals, on the importance of early antimicrobial therapy as well as notify the appropriate hospital and public health agencies. All of these actions will ultimately aid public health in controlling the incidence of pertussis cases, thus ensuring the protection of the general public from this re-emerging respiratory illness.

## Figures and Tables

**Figure:**
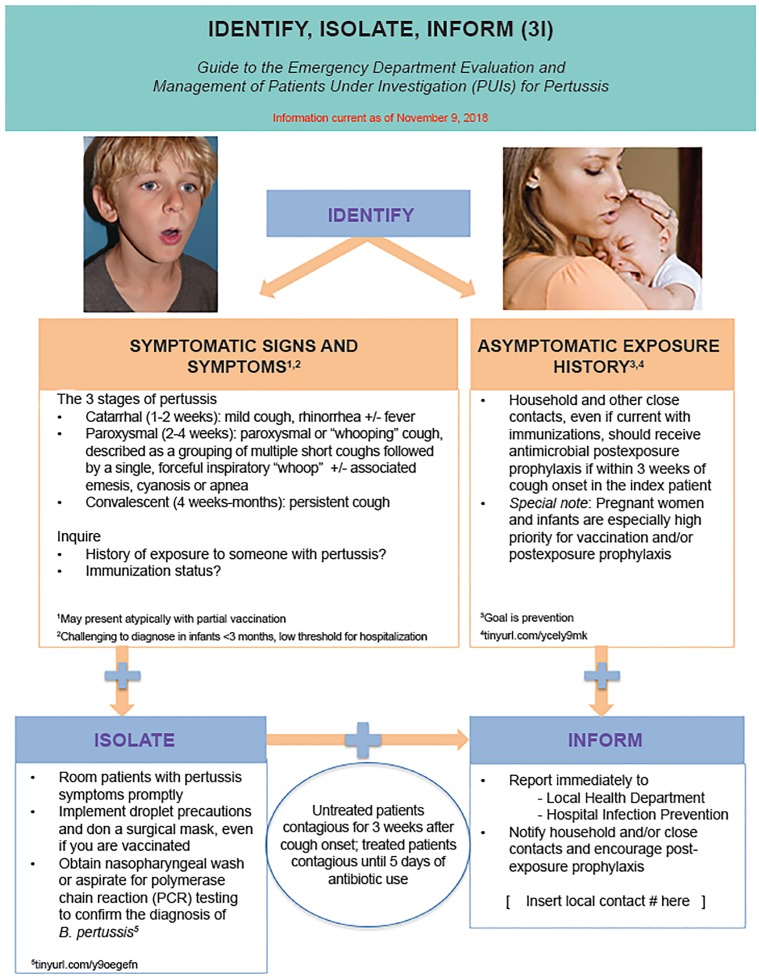
The Identify, Isolate, Inform 3I Tool for Pertussis. The Identify, Isolate, Inform 3I Tool was conceived by Dr. Kristi L. Koenig, County of San Diego EMS Medical Director & Professor Emerita, UC Irvine.

**Table:** Candidates for pertussis post-exposure prophylaxis.

Household contacts	High-risk individuals
Even if asymptomatic and/or current with immunizations, should receive antimicrobial treatment within 21 days of cough onset in the index patient	Infants Women in their third trimester of pregnancy Caregivers or household contacts of infants Anyone who works in or attends a childcare setting
